# Pavlovian conditioning of gentoo penguins (*Pygoscelis papua*) to underwater sound

**DOI:** 10.1242/bio.059425

**Published:** 2022-11-03

**Authors:** Maria S. Rasmussen, Kenneth Sørensen, Malou F. Vittrup, Magnus Wahlberg

**Affiliations:** ^1^ Department of Biology, University of Southern Denmark, Campusvej 55, 5230 Odense M, Denmark; ^2^ Institute for Biosciences, University of Rostock, Albert-Einstein-Str. 3, 18059 Rostock, Germany

**Keywords:** Penguins, Underwater hearing, Anthropogenic noise, Pavlovian conditioning, Foraging

## Abstract

Penguins are known to react to underwater noise, but it is unknown if they make use of sound cues while diving. We tested whether captive gentoo penguins (*Pygoscelis papua*) can pair underwater sounds with food through Pavlovian conditioning. Two seconds after an underwater sound (a 1-4 kHz sweep with a received level of 130 dB re 1 µPa RMS) was played back to one or two unidentifiable penguins, a dead fish was flushed into the water close to the underwater sound source. After 8 weeks of conditioning, one or more individual penguins approached the sound source after sound emission in 78.3% out of 230 trials. In 43 intermixed control trials with no sound preceding the fish, the penguins did not show any reaction in the pre-flush period. In an additional experiment, three identified penguins reacted to the sound in 66.7-100% out of 30 trials, with 0% reactions in five intermixed control trials. Our experiments demonstrate that gentoo penguins can be conditioned to underwater sound and that they associate underwater sounds with food. It is possible that gentoos, as well as other species of penguins, use sound cues while foraging. This may explain why penguins have been observed to react negatively to anthropogenic noise.

## Teaser

**Summary:** Penguins were trained to pair underwater sound with fish. Our results indicate that sounds may serve as important cues for diving penguins and other sea birds.

## INTRODUCTION

Many animals rely on sound during important aspects of their lives, such as orientation, foraging, communication and detection of predators. Sound is not only an important signal modality on land but also in the aquatic environment, where it travels more efficiently than light (Talley et al., 2011). For animals that are secondarily adapted to the aquatic environment, such as marine mammals, aquatic reptiles and aquatic birds, underwater sound offers reliable cues for communication, prey detection and orientation (Thewissen and Nummela, 2008).

The ears of terrestrial animals need adaptations to efficiently detect and analyze sound under water (Sørensen et al., 2022). Marine mammals, such as whales and seals, have acquired a range of auditory modifications to allow for higher hearing sensitivity, larger receiver bandwidth and more acute directional hearing abilities under water than in air (Supin, 2001). Whales, living all their lives in aquatic environments, hear extremely well under water and poorly in air (Kastelein et al., 1997; Liebschner et al., 2005; Mooney et al., 2012), whereas seals, spending time both on land and in water, hear well in both media (Møhl, 1968; Reichmuth et al., 2013; Terhune, 1974).

For aquatic birds, it is less clear as to what extent hearing adaptations are present, and to what degree they utilize sound cues while diving. There are hundreds of species of birds, such as penguins, cormorants and alcids, relying on the aquatic environment for finding food during extended dives. Even though they rely on vision to a large extent to find their prey (Howland and Sivak, 1984), behavioral observations suggest that tactile cues as well as sound may be important when hunting under low light conditions (Castellini and Mellish, 2016; Gremillet et al., 2005; Meir et al., 2008; Ponganis, 2015; Thiebault et al., 2019).

Underwater hearing thresholds have so far been obtained in two species of marine birds, the great cormorant (*Phalacrocorax carbo*; Hansen et al., 2017; Larsen et al., 2020) and the long-tailed duck (*Clangula hyemalis*; Therrien, 2014). The underwater hearing sensitivity in cormorants is comparable to the one of pinnipeds at lower frequencies (1-2 kHz; Hansen et al., 2017; Reichmuth et al., 2013). Furthermore, playback studies show that gentoo penguins (*Pygoscelis papua*), African penguins (*Spheniscus demersus*) and common murres (*Uria aalge*) can react negatively to underwater sounds (Cooper, 1983; Frost et al., 1975; Hansen et al., 2020; Pichegru et al., 2017; Sørensen et al., 2020), even though there is no data on underwater hearing thresholds from these species.

To understand the importance of underwater sound cues to diving birds, we need to assess the ecological benefits of acute underwater hearing. Extreme divers such as penguins are adapted to life at sea, with flippers instead of wings, a dense, waterproof plumage and feet located further back on their body to create less drag while swimming (Lynch, 2007). Penguins are highly reliant on airborne sound for interspecific communication at breeding sites (Jouventin and Dobson, 2018). They also use in-air sound communication during foraging trips (Choi et al., 2017; McInnes et al., 2020). In-air hearing has so far only been assessed in one species, the African penguin, via electrophysiological techniques, showing that their hearing is comparable to that of other similar-sized birds (Wever et al., 1969). It seems, therefore, that penguins are suitable subjects for more carefully addressing the function of underwater hearing in sea birds.

One suitable study species is the gentoo penguin (*P. papua*), performing foraging trips up to 600 km from their colonies and able to stay at sea for almost 25 days (Baylis et al., 2020; Wilson et al., 1996). Gentoo penguins can reach depths of more than 200 m while foraging, during dives lasting longer than 9 min (Ponganis, 2015; Woehler, 2003). They forage on small species of pelagic fish and krill (Croxall et al., 1988). It is not known how gentoo penguins locate prey and navigate at these depths, and whether they utilize sound cues in situations in which vision is restricted, just like marine mammals do.

In marine mammals, the function of hearing can be studied with operant conditioning techniques (Gescheider, 1997; Reichmuth et al., 2013; Schusterman, 1980). For marine birds, operant conditioning training with underwater acoustic stimuli is challenging. Therrien (2014) and Hansen et al. (2017) trained ducks and a cormorant for years. Even though their data clearly showed that their study subjects could hear underwater sounds, the data quality was not as high as what can be obtained from marine mammals. This may be explained by the shorter attention span of birds, leading to much more variability in data, or that sounds are not an important signal cue for birds underwater.

Instead of using operant conditioning for underwater hearing studies, another option is Pavlovian conditioning, also known as ‘classical conditioning’ (Gescheider, 2007; Melfi et al., 2020; Shettleworth, 2012). Here, the animal pairs a stimulus (e.g. an acoustic signal) automatically to a reward (e.g. a fish) after being presented to a stimulus followed by a reward during several trials. When it suffices to present the stimulus to evoke the behavior that is usually observed when presenting the reward, the stimulus is said to have been classically conditioned. When an underwater sound stimulus has been Pavlovian conditioned, we may not only conclude if an animal detects underwater sound, but also if it can pair the sound with other types of sensory cues.

We investigated underwater hearing abilities in gentoo penguins using Pavlovian conditioning to pair an underwater playback signal with a subsequent food reward, while observing the penguin's reactions at the onset of the sound. By establishing a connection between sound and food through Pavlovian conditioning, we demonstrated that penguins are able to associate underwater sound with food.

## RESULTS

### Study A (unidentified subjects)

**Fig. 1.**
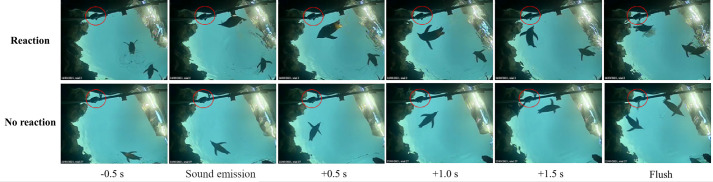
**Examples of responses classified as ‘Reaction’ and ‘No reaction’.** The speaker is in the upper left corner of each frame (indicated with a red ring).

**Fig. 2.**
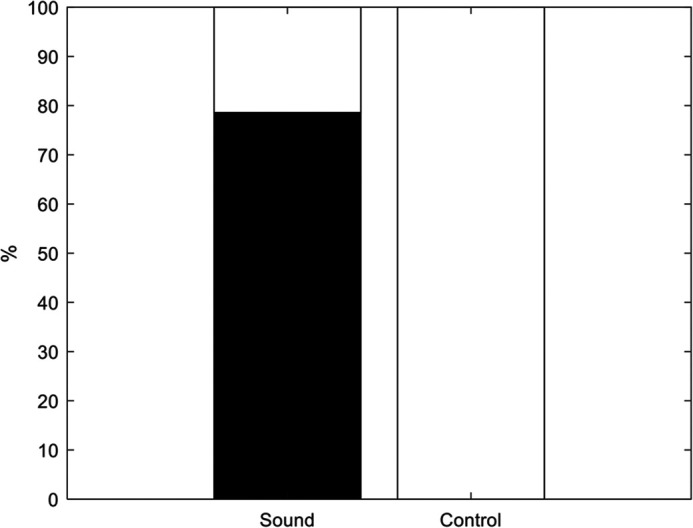
**Results of Pavlovian conditioning in Study A.** The *y*-axis indicates the percentage of trials with reactions (in black). Black is reaction, white is no reaction.

A total of 280 trials with sound stimulus were collected. From these, 230 trials were selected for analysis, omitting trials with penguins in unfavorable locations, as well as trials with disturbances such as porpoising penguins (Table 1). In addition, 50 control trials with no sound playback were randomly included, out of which 43 were selected for analysis. The control trials were judged by the same criteria as in the trials with sound stimulus. No reactions were observed in any of the control trials. Examples of a ‘Reaction’ and ‘No reaction’ for a sound trial are illustrated in Fig. 1. The distribution of ‘Reaction’ and ‘No reaction’ trials were significantly different for acoustic trials compared to control trials (Fig. 2, χ-square test, d.f.=2, *P*<0.001). There was no significant difference between the gradings of the 230 trials of Study A between the three observers (Kruskall–Wallis test, d.f.=2, *P*>0.05). The observers indicated no reaction in all 43 control trials. All observers agreed there was a reaction in 133 out of 230 trials (58%), and in another 47 trials, two of the three observers reported a reaction (resulting in at least two observers reporting a reaction in 78% of the trials). In 34 of the trials, one observer reported a reaction (15%), and in just 16 of the trials, no observer reported a reaction (7%).

**Table 1.**
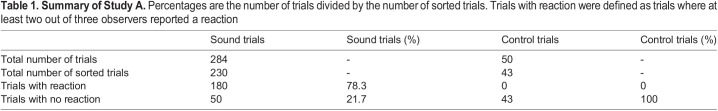
**Summary of Study A.** Percentages are the number of trials divided by the number of sorted trials. Trials with reaction were defined as trials where at least two out of three observers reported a reaction

### Study B (identified subjects)

In Study B, 30 trials with sound were collected. From these, 24 were selected for analysis using the same criteria as in Study A. Only trials where one out of three identifiable penguins could be tracked were used. In three of these trials, two identifiable penguins could be tracked, so the total number of observed penguin reactions were 27 (Table 2). In addition, five control trials with no sound emission were randomly included. All control trials were graded ‘No reaction’ for all observers (Table 2). The distribution of ‘Reaction’ and ‘No reaction’ for the sound trials of all three birds were significantly different from the control trials (one-tailed unpaired Student's *t*-test, *P*<0.001). There was no significant difference between the gradings by four observers of the 27 trials of Study B (Kruskall–Wallis test, d.f.=2, *P*>0.3). If we regard the penguin as reacting by at least three observers, the three individuals were reacting in 67-100% of the trials (Table 2).

**Table 2.**
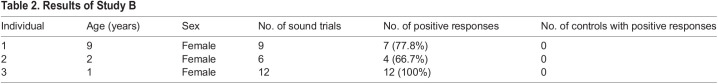
Results of Study B

### Comparison of Study A and B

In Study A, 78.3% of the signal trials were categorized as ‘Reaction’ (with at least two observers reporting a reaction) versus 21.7% of the trials in which there was no reaction. For Study B, there was on average 70.3% ‘Reaction’, and 28.7% ‘No reaction’ (Tables 1 and 2).

## DISCUSSION

A minimum of three individual gentoo penguins were Pavlovian conditioned to approach an underwater speaker after an underwater sound stimulus was emitted. In Pavlovian conditioning terminology, the sound stimulus was the neutral stimulus and fish was the conditioned stimulus. The unconditioned response was to approach the fish. After hundreds of trials, the sound stimulus became the conditioned stimulus, causing a conditioned response (approaching the speaker as a response to sound). The responses were subsequently judged by three independent observers.

Our experiments were made in a public facility, making it difficult to negotiate controlled experimental conditions. We cannot rule out several consequences on the quality of the data obtained here due to these conditions. First, we could not control for which penguins were present in the pool during each sound exposure. Therefore, it is possible that non-focal penguins watched other penguins respond to the sound, and in that way became conditioned to the sound faster than what would otherwise have been the case. Second, individuals who had only been exposed to the sound a few times or not at all may have been in the pool at the time of data collection. These individuals would most likely not have reacted to the sound, as they had not been conditioned to it. Third, depending on individual penguins’ participation in the trials, they would have received different amounts of food during the experiments, which may have affected their motivation to participate in subsequent trials. As confounding for the data these three issues may be, all of them strengthen the general conclusion of this study that penguins can be conditioned to underwater sound. Also, during some of the trials, there was only one penguin in the pool, showing acoustic conditioning behaviours.

Pavlovian conditioning is a standard method to learn how animals make new connections between different stimuli. The fact that underwater sound was transformed from a neutral to a conditioned stimulus strongly indicates that penguins can learn to associate underwater sound cues with food. In addition to earlier experiments showing that penguins react to underwater sound (Frost et al., 1975; Pichegru et al., 2017; Sørensen et al., 2020), our study suggests that penguins can make use of underwater sound cues.

To extract information from the underwater soundscape, it is not only important for an animal to detect sounds, but also to pinpoint the direction of the sound source. Whereas marine mammals have excellent directional underwater hearing abilities (Au, 1980; Supin, 2001; Terhune, 1974), no conclusive experiments have been conducted on marine birds in this respect. However, the fact that underwater sound elicits directional responses both for aversive (Sørensen et al., 2020) and attractive (this study) signals makes it presently impossible to rule out the presence of directional hearing abilities in penguins, and calls for more experimentation on this subject. However, the physiological mechanism of how penguins would be able to pinpoint the direction to the underwater sound source is presently not understood. In our study, penguins would sometimes move their heads from side to side while diving, perhaps to search for the food visually, or for refining interaural cues for directional hearing, as is often observed in terrestrial animals (Wallach, 1940).

There are several underwater sound cues that could be of interest for diving penguins to orient themselves and to find prey. The underwater soundscape comprises abiotic and biotic sounds (Urick, 1983). By tapping into signals stemming from reflections from the sea surface and sea floor, as well as from coasts, penguins may be able to determine their depth in the water column, as well as their distance to the coast, the composition of the sea floor and many other features that help them to spatially orient themselves between resting and feeding sites. Also, many fish species produce sounds, both actively for communication and passively during other activities, such as feeding or spawning (Ladich, 2019). The ability to detect and pinpoint the direction to fish sounds may reduce the time penguins use to search for prey and thereby gain foraging efficiency. In addition, underwater vocalizations were recently reported during foraging in multiple penguin species (Thiebault et al., 2019). Such sounds, intentionally or unintentionally made by nearby conspecifics while hunting, could be used as cues for finding prey and/or coordinate foraging strategies. Finally, if penguins can learn to avoid the underwater calls of some of their main predators, such as killer whales *(Orcinus orca)* and leopard seals (*Hydrurga leptonyx*; Richlen and Thomas, 2008; Rogers, 2014), as, for example, harbour seals do (Deecke et al., 2002), they may more easily avoid ending up as prey.

The hypothesis of penguins using underwater sound cues while diving is supported by the fact that e.g. emperor penguins (*Aptenodytes forsteri*) perform extremely deep foraging dives, reaching depths of more than 500 m (Meir et al., 2008; Ponganis, 2015). Such depths are well below the euphotic zone (Talley et al., 2011), so no sunlight is present. Underwater sound cues could help penguins to orient themselves and to find prey in complete darkness.

Studies suggests that some aquatic birds other than penguins also use underwater hearing. Great cormorants (*P. carbo*) and long-tailed ducks (*C. hyemalis)* can detect auditory cues while diving (Hansen et al., 2017; Therrien, 2014). It is not known whether cormorants and diving ducks can pinpoint the direction to underwater sound sources and relate sound cues with prey. For other aquatic bird species, such as auks and gannets, in-air hearing sensitivity has only been measured in three species so far (Crowell et al., 2016; Mooney et al., 2020). Compared to marine mammals, there is still a lot to learn about the importance of sound cues for aquatic birds.

The three individual penguins participating in Study B were the three youngest gentoo penguins in the enclosure. The youngest penguin participated in more trials than the other two, and it received the highest grading. The second youngest penguin, however, got the lowest grading. More data are needed to determine how age affects Pavlovian conditioning for underwater sounds in penguins. Also, any other differences in the response of the penguins to underwater sounds across sex, ‘personality’ and other individual features have to await further experimentation to be determined.

Some of the penguins used in the first experiment were never conditioned to sound. Also, the penguins that were conditioned did not respond to the sound in every trial, even after conditioning was completed. There may be several reasons why the conditioning did not work on all individuals, and why conditioned individuals did not respond every time the sound was presented. There may be individual differences between penguins, and each penguin may not be motivated by food in each trial. The experiments were made in a public facility, making it difficult to control the experimental conditions. It is therefore not possible to discern which factors may have influenced the performance of the penguins during the trials. In the second experiment, in which individual penguins could be recognized, we observed conditioning in all three test subjects and in the majority of all trials.

As penguins seemingly have sensitive underwater hearing abilities, this calls for more work examining how they are affected by human-induced noise. Rapidly increasing noise levels from ship trafficking have coincided with an 85% population decline of African penguins (*S. demersus*) around St. Croix Island since 2016 (Pichegru et al., 2022). Also, underwater blasting from seismic activities have shown to induce strong avoidance behavior of African penguins and displaced colonies from their preferred foraging area within 100 km of the operational areas (Pichegru et al., 2017). In extreme cases, such seismic activities could be fatal to colonies within close proximity of the blasting areas (Brown and Adam, 1983; Cooper, 1982). If penguins use sounds as cues while foraging (as indicated in our study), they may also be affected by hearing threshold changes and masking, just like whales and seals (Gordon et al., 2003; Finneran, 2015; Weilgart, 2007).

Our data suggest that acoustic cues may be more important for penguins (and perhaps also other aquatic birds) than previously expected. If underwater sounds are important cues for penguins, this may alter our understanding for their sensory ecology. The fact that penguins react to, and are Pavlovian conditioned to, underwater sound also calls for us considering these birds when effects of human-induced noise is evaluated on wildlife.

## MATERIALS AND METHODS

Experiments were conducted in the penguin enclosure of Odense Zoo, Denmark. The 175 m^3^ pool has a surface area of 50 m^2^ and a depth of 3.5 m, filled with artificially tempered and filtered saltwater with a temperature of 6±1°C and salinity 28‰. In-air temperature was 2-7°C. The irregularly shaped concrete pool walls efficiently diffused sound reflections, adequate for underwater acoustic experiments.

The enclosure housed three species of penguins: 20 king penguins (*Aptenodytes patagonicus*), 12 northern rockhoppers (*Eudyptes moseleyi*) and 11 gentoo penguins (the focal species of this study). The gentoo penguins participating in the experiments, both females and males, ranged in age from less than 1 year to 26 years. One of the penguins used in these experiments had previously been used in a study on the penguins' reaction to underwater sounds (Sørensen et al., 2020).

**Fig. 3.**
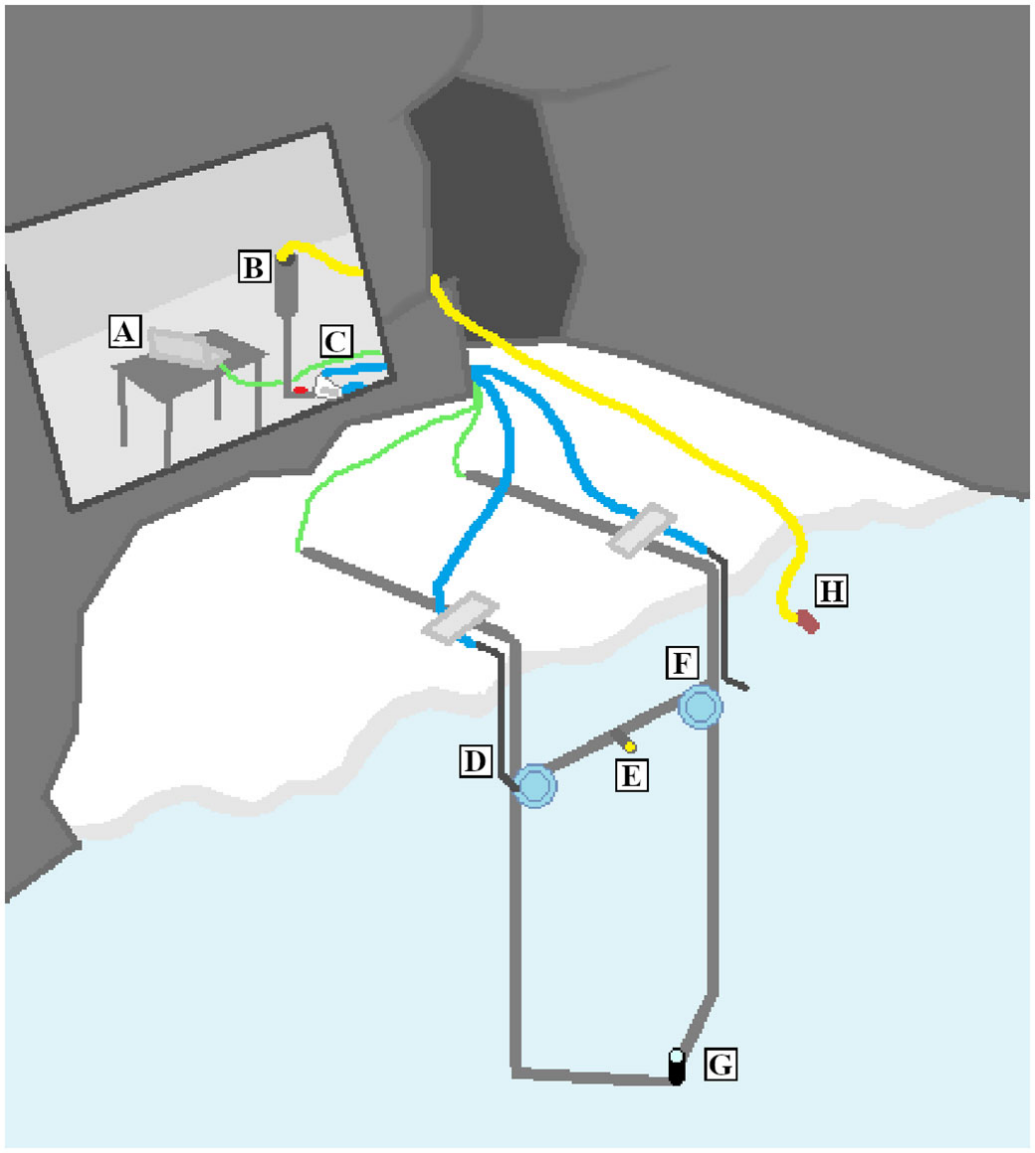
**Setup used for classical conditioning of penguins to underwater sound.** The experimenter was positioned by the computer without direct contact with the penguins during trials. A, laptop; B, PVC pipe cannister for water flush; C, manual valve; D, hose and PVC pipe for flushing fish; E, trial light; F, loudspeaker; G, underwater camera; H, bilge pump.

The experimental setup (Fig. 3) consisted of a PVC rig with an underwater trial light (indicated by D in Fig. 3), a fish flush (E), a loudspeaker (F; University Sound UW30, Lubell, OH, USA) and an underwater camera (G; Divers Pro Fish-eye 10-021, LH-video, Kolding, Denmark). The video camera was positioned at a depth of 3 m, directed upwards towards the loudspeaker and fish flush terminal at a depth of 1 m. In Fig. 3, two loudspeakers and two hoses for flushing fish are shown. In this study, only the left speaker and fish flushing hose was used. The second loudspeaker and fish flushing system were intended for subsequent studies on directional hearing. A laptop computer (A) equipped with LabView (National Instruments, Austin, TX, USA) was connected to the loudspeaker (F) via a 12 V amplifier to administer the sound stimulus. The laptop was also connected via an Elgato (Munich, Germany) video capturing device to an underwater camera. The fish flush consisted of a 9 l water reservoir (B), a valve, a 3 cm diameter plastic hose and a PVC pipe ending 5 cm above the loudspeaker. Next to the valve there was a plug, which could be removed to insert a fish into the hose (C). Between trials, the water reservoir was filled using a bilge pump (H), connected to the reservoir via another hose.

The operator was positioned with the fish flush reservoir and valve, as well as the laptop and additional electronics, behind a wall 3 m from the pool and out of sight of the penguins. The operator used a custom-made LabVIEW (National Instruments) program and video software (Elgato) on the laptop to administer sound emissions, fish flushing and refilling the water reservoir. The trial light was turned on at the start of each session and turned off when the session ended, to indicate to the penguins that an experiment was ongoing. During a session (which lasted 25-60 min), the experimenter observed the penguins in the water using the underwater video camera to choose the right time to play the sound stimulus, based on the following criteria: (1) the penguin had to swim approximately 1 m below the surface; (2) the penguin had to be within, and preferably in the center of, the camera's field of view; (3) the penguin had to swim calmly through the water; and (4) no penguins were swimming fast and/or porpoising in the pool.

When 2 s had passed after a stimulus had been played out, an indicator lamp was lit on the computer screen, indicating for the operator to flush the fish. The 2 s time delay was introduced to allow the experimenter to evaluate the penguins' reactions to the stimulus without confounding their reaction to flushing sounds, water and bubbles coming out from the pipe delivering the fish. After each trial, the valve was closed, the reservoir was filled, and the system was charged with a fish, to be ready for the next trial.

The reinforcer was either a half sprat (*Sprattus sprattus*) or a third of a capelin (*Mallotus villosus*). Both species are part of the regular diet of the penguins in the zoo, and they were selected in cooperation with the zookeepers responsible for the penguins. The total amount of food used in each experiment was about 200 g. When split between the penguins involved in the trials, each penguin only received a small amount of its daily food (about 25 g per penguin) during the experiments. There were on average 2-3 min between each trial.

**Fig. 4.**
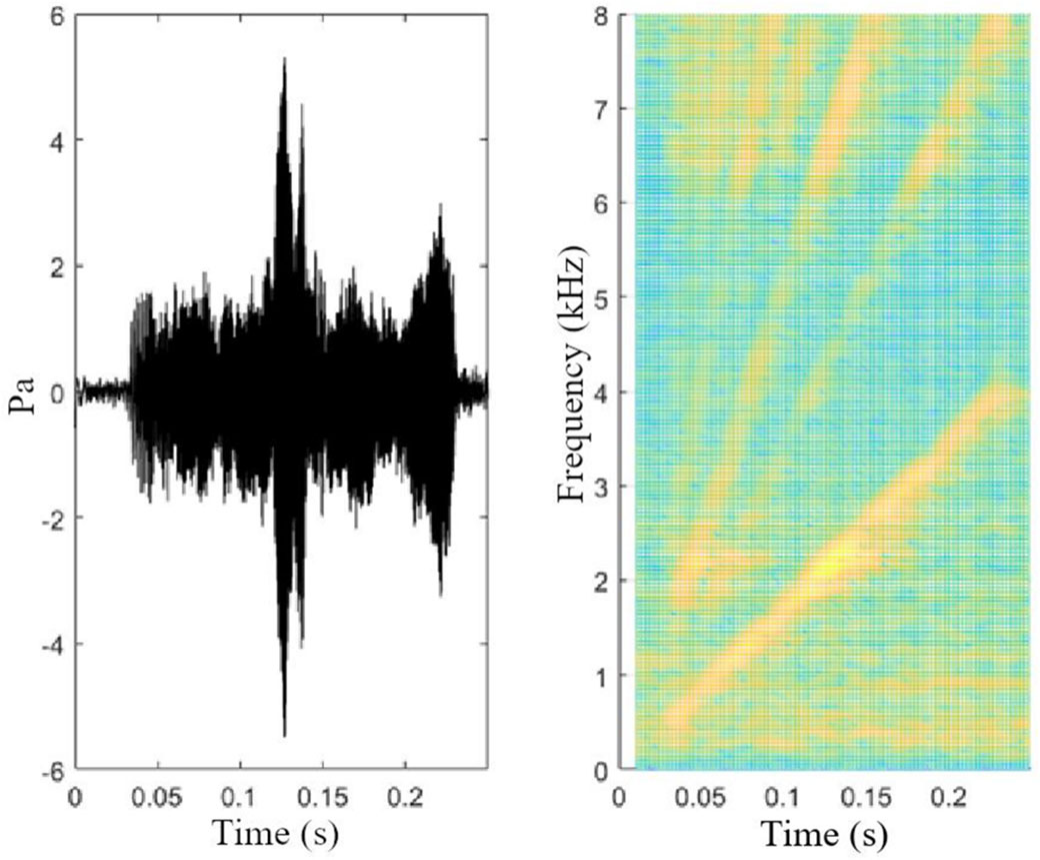
**Stimulus oscillogram and spectrogram of stimulus used during acoustic conditioning.** Sampling rate 48 kHz, 16 bits. Spectrogram with fast Fourier transform (FFT) size 1024, 87% overlap, Hanning window.

The stimulus was regularly measured with a SoundTrap HF300 data logger (OceanInstruments, New Zealand) 1 m in front of the loudspeaker. The recorded stimulus signals (Fig. 4) were band-pass filtered in MATLAB, and the received level was measured as the root mean square (RMS) of the 95% energy duration (see Madsen and Wahlberg, 2007 for details). The signal sound level was 130 dB re 1 µPa and varied with less than 3 dB. This sound level was chosen based on the study by Sørensen et al. (2020), who played back sound to the same group of penguins studied here. The results indicated that the chosen sound level was clearly audible to all penguins in the pool at the given frequencies.

Prior to data collection, the penguins were conditioned to the sound stimulus over the course of several months (December 2020 – March 2021), with pauses of a few weeks in between bouts of sessions. A total of more than 1000 trials distributed over more than 50 sessions were conducted using the same protocol as outlined above, with a few adjustments. In the initial conditioning process, there was no delay between the emission of a sound stimulus and the flush of a fish. A few months later, a delay of 1 s was introduced, and a few days before the data collection started, the delay was increased to 2 s.

During data collection, each session consisted of 35 sound trials and three to seven control trials (with no sound) randomly intermixed into each session, and a maximum of one session every day. For control trials, no sound was played back, but when the penguins were positioned correctly according to the criteria described above, the flush was operated with no fish inside 2 s after the ‘no sound’ presentation. Each session lasted 20-30 min, and the time between trials was less than 2 min.

Data for the first study (Study A) were collected from 10 March to 1 April 2021, over 10 days, with one session per day providing 230 usable trials. In Study A, it was not possible to discern which individual penguin was participating in each trial. All penguins were consequently pooled for data analysis.

After Study A, conditioning sessions continued, with three to seven sessions per week. A GoPro camera was mounted in the air above the setup pointing towards the loudspeaker and the fish flush to identify which penguins participated in the experiment (discernable from colored plastic wing bands). On 12 May 2021, data for three known individuals were collected (Study B). In Study B, 30 signal and five control trials were made in one session. Due to human error or too much disturbance from non-focal penguins in the pool, five of the 30 trials were excluded from the analysis. Some trials included more than one penguin; therefore, a total of 27 reactions were investigated.

In some trials, there was only one penguin present in the pool, whereas in other trials, there were several penguins present. Due to this being a public facility, it was not possible to control for how many penguins were in the pool during the experiments.

The underwater video recordings from all trials were sorted in terms of quality before analysis. Trials were excluded for either of the following reasons: (1) more than two penguins were visible in front of the loudspeaker; (2) the sound was played back while the penguin's head was above the water surface; (3) the sound was played back while the penguin was not in front of the camera; and (4) high levels of electric or ambient noise and other technical issues.

The criteria in Table 3 were used in Studies A and B for evaluating the birds’ reactions from the onset of the playback sound and until 2 s had passed. The distribution of reactions (categorized as ‘Reaction’ and ‘No reaction’) was compared for sound and control trials using a χ-square test.

**Table 3.**
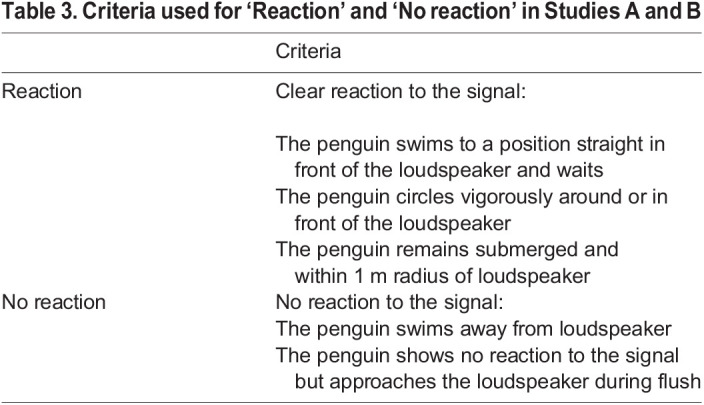
Criteria used for ‘Reaction’ and ‘No reaction’ in Studies A and B

To make the results as objective as possible, data from Studies A and B were analyzed with three and four observers, respectively. All observers were students or scientists with previous experience grading behavioral responses from playback studies on marine animals. Both studies were graded using the same criteria. The classification of the observers was compared using a Kruskall–Wallis test. During the analysis of trials from Study B, the focal penguin being observed was marked with a red ‘X’ in the video recordings to ensure that all observers focused on the same bird. In this part of the study, all gentoo penguins within the underwater camera view were included, as long as they were identifiable with the in-air GoPro camera.

Animal experimentation was conducted under a permit to the University of Southern Denmark DVO (approval number: 2021/04), acknowledged by the Danish Animal Experimentation Inspectorate.
